# Bibliographical

**Published:** 1887-08

**Authors:** 


### BIBLIOGRAPHICAL.

A SYSTEM OF DENTAL SURGERY. By SIR JOHN TOMES, F. R. S. Third edition, revised and enlarged. By CHARLES S. TOMES, M. A., F. R. S. With 292 illustrations. Philadelphia: P. Blakiston, Son & Co. 1887.

There is no dental text book which occupies a higher place in the estimation of our English confrères than does the work under notice. First issued in 1859, it immediately leaped into professional favor, and took its place at the head of professional literature, a position which it still maintains and seems likely long to hold. The high favor with which it is regarded in England is due to the many excellencies of the work itself. John Tomes has always borne the reputation of a man who never made an assertion until he was certain of his facts. His name has not often appeared in dental literature, but when it has the student always felt assured that the subject matter had been well considered. He never jumps at conclusions, nor rests assertions upon a few hurried observations. As a consequence he has not, like many so-called investigators, been compelled to retract one day what he had confidently asserted the previous one. Nor has he misled his readers, teaching them false doctrines, and so destroying the confidence which had been reposed in him; if his methods were sometimes called slow, they possessed the inestimable advantage of being sure.

In the preparation of the new edition by the younger Tomes, all the leading characteristics of the earlier editions have been retained, while the whole has been modernized and the newer observations and discoveries of later years are judiciously incorporated. That this was a task of no small proportions the careful student must be aware, for duly to consider the almost numberless theories and records of the many who have made contributions to scientific dental literature, to select the wheat and leave the worthless chaff, to condense and to harmonize that which seems incompatible, and to reduce to a system the many jarring inconsistencies of zealous but at times injudicious writers, was a labor that but few are quite competent to perform. Mr. C. S. Tomes has accomplished it admirably.

There is another virtue which the book possesses. Instead of being padded out to unseemly proportions by borrowing from dental manufacturers the wood cuts of their simplest appliances, and endeavors to enlarge the book by a kind of covert advertising, it contains little that is not indispensable to a thorough comprehension of the subject. We would that some American publishers of dental works would copy these good qualities. The revised edition should meet with a large sale in this country.

A TREATISE ON DIPHTHERIA. Historically and practically considered, including Croup, Tracheotomy and Intubation. By A. SANNE, Docteur en Médicin, Chevalier de la Légion de Honneur, etc., etc. Translated, annotated, and the surgical anatomy added by Henry Z. Gill, A. M., M. D., LL. D. St. Louis: J. H. Chambers & Co. 1887. Pp. 656.

Medicine has made wonderful advances of late in the methods of the treatment of many diseases, but in no field perhaps have more important discoveries been made than in tracheal affections. In fact, the scientific methods of combating diphtheria are almost entirely modern, and the practitioner who is not familiar with the late medical literature upon this subject is quite unfit for his duties. Yet the articles published upon this most important subject are scattered through the journals, or exist in the form of monographs, which are not easily obtained. The admirable and exhaustive work of Dr. Sanné has brought into one volume and within the reach of every practitioner all the experience of the past and the knowledge of the present, and may be regarded as a complete treatise upon its subject. There are very few—if any—fields of medicine that have received such a complete exposition and are so thoroughly and exhaustively considered as is the one chosen by our author. The physician who is the possessor of Sanné needs no other reference or text book.

The first part is given up to the surgical anatomy of the pretracheal region, with reference to tracheotomy in children, and so numerous and graphic are the illustrations that they afford a complete study of the parts. Then the pathology of all the varying conditions in diphtheria is carefully considered, its treatment, both medicinal and surgical, minutely explained, and all the varying sequelæ pointed out. It would be impossible within the limits of a journalistic notice to fully speak of all the merits of this most complete text book in medicine. It must suffice to say that it is one which the general practitioner cannot afford to be without, for it is the summing up, the complete crystallization of all the knowledge of the present day, of the matter of which it treats.

J. H. Chambers & Co. have furnished to medicine books of great value, but we believe that neither they nor any other publishers have offered anything of greater importance in any special field of medicine than is this work in its own domain.

Earth as a Topical Application in Surgery. Being a full exposition of its use in all the cases requiring topical application admitted in the men's and women's surgical wards of the Pennsylvania Hospital, during a period of six months, in 1869. By Addinell Hewson, M. D. Second edition, with four photo-relief illustrations. Philadelphia: The Medical Register Co. 1887.

Not content with issuing one of the best of the medical weeklies, *The Medical Register*, its publishers have entered upon the work of furnishing to the profession a series of useful and popular medical books, and we are in receipt of two neat volumes from their press, both of which are of an exceedingly practical nature—hand-books for every day reference. The character of medical literature has materially changed within a few years, and instead of the elaborate and prolix treatises covering almost the whole domain of medicine, and appearing only at long intervals, we now have brief monographs upon some specific subject, written, perhaps, in a hurried manner and possessing little claim to literary excellence, but bearing all the marks of freshness and earnestness exhibited by records direct from the laboratory or bedside. Such books should be the companions of students and practitioners engaged in special observations, for they contain the data with which earnest men may compare their own observations, and thus arrive at a definite conclusion concerning any proposed method of practice.

There is, for instance, scarce an intelligent man who has not at some time been impressed with an idea of the virtues of fresh earth in the treatment of wounds and cases of poison inoculation. He has in boyhood listened to tales of its curative virtues, and in his more mature years has, perhaps, himself observed such instances. The book under notice contains the record of ninety-three cases of hospital treatment by earth dressings, with notes and comments, the record of microscopical examinations, and an exposition of the *modus operandi*. Every physician knows the value of earth closets in deodorizing and disinfecting evacuations, and it was this efficiency which first suggested its use in the treatment of ulcers and putrefactive inflammations. Some of the cases detailed are remarkable, and in this day of antiseptic treatment should be studied by every practitioner. Price, $1.00.

WHAT TO DO IN CASES OF POISONING. By WILLIAM MURRILL, M. D., F. R. C. P. First American from the fifth English Edition. Edited by Frank Woodbury, M. D. Philadelphia: The Medical Register Co. 1887.

There is no emergency for which the physician needs to be more thoroughly prepared than for a case of poisoning. In such instances an instant of time is sometimes worth a human life. There is no leisure for an examination of standard text books, nor for the compounding of an elaborate prescription. Every one about has usually lost his presence of mind, and is in such a state of excitement and confusion that he is an absolute embarrassment. At such a time the physician should be competent to assume entire direction, and, in the absence of usual remedies, be prepared to utilize whatever the vicinity affords for relief. Unless he is possessed of the necessary information he cannot do this. His "dose-book" is at home, and his table of antidotes is with it. If he have not the knowledge which may be obtained by the study of such books as this, the physician himself is in the way. Let him obtain it and master its contents, and he will then be armed for any case of poisoning which he may be called upon to attend.

PRACTICAL LESSONS IN NURSING.

*I. The Nursing of and Caring for the Nervous and the Insane.* By CHARLES K. MILLS, M. D.

*II. Maternity, Infancy and Childhood.* By JOHN M. KEATING, M. D.

*III. Outlines for the Management of Diet.* By EDWARD TUNIS BRUEN.

Philadelphia: J. B. Lippincott Company. 1887. Price, $1.00 each.

These are the first three volumes of a series of hand books devoted to that most important therapeutical agent, nursing and diet. Every thinking physician knows that the welfare of his patient depends quite as much upon the nursing and care which is given as upon the medicines administered, and very often the latter are but secondary and of little importance as compared with the former. There is an unsuspected amount of ignorance concerning these things in households of supposed intelligence, and the medical attendant too often is required, not only to direct what shall be the diet of the sick, but to be able to give instructions in the proper preparation of the food. He must not alone direct what shall be the attentions administered, but illustrate them to the attendants, for it is not often that the trained nurse will be at his command. The physician, then, must possess the information which these books convey. If in addition he could place the proper one in the hands of friends of the sick, he would enhance his own reputation and be assured of better care for his patients.

Every expectant mother, for instance, and every one who has the care of children, should possess a copy of "Maternity, Infancy and

Childhood," and should make a careful study of it. Dr. John M. Keating is an authority upon the subject of which he speaks, and there is so much of information condensed within a small compass that we almost wonder how any intelligent woman can undertake the care of the child given her without a study of the subject, such as may be obtained from this little volume.

The other volumes are of equal import in their own field. They are adapted, not alone to the professional man, but to the apprehension of every one who cares to know anything of the laws which govern the human system in health or disease.

THURLY TIGHE; OR, THE LIFE OF A STUDENT. By FELIX WEISS, L. D. S., author of "Vernon Galbray," etc., etc., etc.

We have spoken of this capital story when it was in course of publication as a monthly supplement to *The Dental Record* of London. We can only compliment the author of this entertaining and instructive dental tale, and hope that this will not be the last book from his pen which we shall have the pleasure of reading.

TRANSACTIONS OF THE ODONTOLOGICAL SOCIETY OF NEW YORK FOR 1886. Philadelphia: The S. S. White Dental Manufacturing Company.

This is another volume of a valuable series. The transactions of this society, handsomely issued as they are by the publishers, form a valuable addition to any dental library.

TRANSACTIONS OF THE NEW JERSEY STATE DENTAL SOCIETY FOR 1884, '85 AND '86.

The New Jersey State Dental Society is one of the most active of all the organizations in this country. It includes within its membership men well known to dentistry, clear and vigorous thinkers and writers, and the record of its doings is of interest to all progressive dentists.

*The Management of Pulpless Teeth.*

This is a capital monograph, published by the Chicago Odontological Society, a copy of which should be in the hands of every progressive dentist.

*Twentieth Annual Report of the Trustees of The Peabody Museum of American Archæology and Ethnology.*

*Baked Beans.* A serio-humorous medical paper. By EPHRAIM CUTTER, A. M., M. D. Reprinted from *Albany Medical Annals.*

*Transactions of the Louisiana State Dental Society for* 1886.

*Report of the Proceedings on the Occasion of the Presentation of a Testimonial to Dr. W. H. Waite,* at the annual meeting of the Midland Branch of the British Dental Association, held in Chester, April 29, 1887.

*Transplantation of a Rabbit's Eye into the Human Orbit.* By CHARLES H. MAY, M. D. Reprinted from *The Archives of Ophthalmology.*

*Importance and Value of Experimental Research.* By N. SENN, M. D. Reprinted from *The Western Medical Reporter.*

*Some Considerations Concerning Cancer of the Uterus,* especially its palliative treatment in the later stages. By ANDREW F. CURRIER, M. D. Reprinted from *The New York Medical Journal.*

*Persistent Pain After Abdominal Section.* By JAMES B. HUNTER, M. D. Reprinted from *Gynecological Transactions.*

*The Uses of Massage in Medical Practice.* Translated from the German of Reibmayr. By BENJAMIN LEE, A. M., M. D., Ph. D.

*Prescription Writing.* By CHARLES H. MAY, M. D. Issued for the use of his Quiz Classes.

*Feeding Patients Against the Appetites.* By EPHRAIM CUTTER, M. D., M. M. S. Reprinted from *The Medical Register.*

*President's Annual Address* at the Midland Branch of the British Dental Association. By FRED. BULLIN, J. P., L. D. S., England.

*Pelvic Inflammation;* or, Cellulitis *versus* Peritonitis. By THOMAS ADDIS EMMETT, M. D. Reprinted from *Gynecological Transactions.*

*Congenital Occlusion of the Posterior Nares.* By ALVIN A. HUBBELL, M. D. Reprinted from *The Buffalo Medical and Surgical Journal.*

*Third Annual Report of the Board of Dental Examiners of Iowa.*

*Fourth Annual Report of the Board of Dental Examiners of Iowa.*

*Report of the Board of Dental Examiners of California.*

